# Risk factors of presenile nuclear cataract in health screening study

**DOI:** 10.1186/s12886-018-0928-6

**Published:** 2018-10-11

**Authors:** SW Nam, DH Lim, KY Cho, HS Kim, K Kim, T-Y Chung

**Affiliations:** 1 Department of Ophthalmology, Samsung Medical Center, Sungkyunkwan University School of Medicine, Seoul, South Korea; 2 Department of Preventive Medicine, Graduate School, The Catholic University of Korea, Seoul, South Korea; 3 Biostatistics and Clinical Epidemiology Center, Research Institute for Future Medicine, Samsung Medical Center, Seoul, South Korea; 4 Department of Ophthalmology, Samsung Medical Center, #81 Irwon-ro, Gangnam-gu, Seoul, 06351 South Korea

**Keywords:** Presenile cataract, Health screening test, Scheimpflug image, Smoking, Exercise

## Abstract

**Background:**

To identify risk factors for the development of presenile nuclear cataract in health screening test.

**Methods:**

The cross sectional study included a total of 532 eyes of 266 participants aged 30 to 49 years of Samsung Medical Center from February 2013 to April 2015. Presence of nuclear cataract was defined when the log MAR visual acuity with correction was greater than or equal to 0.2 and one or more of the following were met: Pentacam Nuclear Staging (PNS) grading score ≥ 1, average value of nuclear density ≥ 15%, maximum value of nuclear density ≥ 30%. Possible risk factors were obtained from blood tests and questionnaires of a health screening test of Samsung Medical Center. Association between nuclear cataract and risk factors was investigated using univariate and multivariate logistic regression analysis by generalized estimating equation (GEE) models.

**Results:**

Five factors were significantly associated with presenile nuclear cataract: current smoking [odds ratio (OR) = 2.80, 95% confidence interval (CI), 1.10–7.12, *p* = 0.0310], non-exercise and high amount of daily physical exercise (OR = 3.99, 95% CI, 1.27–12.52, *p* = 0.0178; OR = 2.92, 95% CI, 1.38–6.22, *p* = 0.0053), asthma (OR = 8.93, 95% CI, 1.12–71.15, *p* = 0.0386), tuberculosis (OR = 4.28, 95% CI, 1.36–13.50, *p* = 0.0131), and higher total iron binding capacity (OR = 1.01, 95% CI, 1.00–1.02, *p* = 0.0059).

**Conclusions:**

Presenile nuclear cataract is related to current smoking, non-exercise or high amount of physical exercise, asthma, tuberculosis, and iron deficiency status. The association of non-exercise group and presenile nuclear cataract seems to be related to co-morbidity. Patients with asthma, tuberculosis, or iron deficiency anemia are recommended to receive frequent ophthalmic examination to detect cataract.

**Electronic supplementary material:**

The online version of this article (10.1186/s12886-018-0928-6) contains supplementary material, which is available to authorized users.

## Background

Cataract, caused by degenerative changes in the lens, is a major cause of blindness globally, and often occurs after 50 years of age [1]. The Lens Opacification Classification System III (LOCS), the most commonly used grading system for evaluating cataract, grades cataract by nuclear color and opacity, cortical opacity, and posterior subcapsular opacity [2].

Pentacam Nucleus Staging (PNS) using Pentacam Scheimpflug (Oculus, Wetzlar, Germany) [3] images is a quantitative method of measuring nuclear cataract that provides average and maximal lens density [4]. In the previous study, we suggested that Pentacam imaging system is effective in screening cataract patients and has the potential to be applied in health examination [4]. The correlation between PNS and LOCS III has been revealed in many studies [5–7], especially in nuclear cataract [8]. PNS detects early nuclear cataract and quantitatively analyzes nuclear cataract [6].

Presenile cataract refers to onset before the age of 50 years [9]. According to a previous study, posterior subcapsular cataract related with atopy is the most common type of cataract in presenile age [9]. However, early diagnosis of other types of cataract including nucleosclerosis in presenile age is important to make a clinical decision of cataract surgery and prevent progression of cataract. Unlike senile nuclear cataract, the risk factors of presenile nuclear cataract is well not known.

The purpose of the present study is to detect and reveal the risk factors of presenile nuclear cataract diagnosed by Pentacam image in the health screening test.

## Methods

The study population included 4605 consecutive participants undergoing screening cataract using Pentacam Schiempflug imaging as part of routine health check-up examinations at the Center for Health Promotion of Samsung Medical Center in Seoul, Korea from February 2013 to April 2015. Of these, 532 eyes of 266 participants aged 30 to 49 years were included. All participants had no history of ocular trauma, laser treatments, or ocular surgeries. Participants diagnosed with any ocular disease except age-related cataract were excluded. If a participant had more than one Scheimpflug image during the study period, only the last Scheimpflug image was included.

The Institutional Review Board of Samsung Medical Center (Seoul, Korea) approved the study protocol (IRB File Number: 2015–06-050). The Institutional Review Board of Samsung Medical Center exempted the requirement for informed consent because the study was based on retrospective analyses of de-identified existing administrative and clinical data.

### Health screening examination

Over 1200 predictor variables were obtained by health screening examination. According to previous reports [10–16], 42 of these predictors known to be associated with cataract were selected for the analysis: age [14], sex [17], history of smoking [18], alcohol consumption [19], physical exercise [20], diabetes [21], asthma [22, 23], tuberculosis [24], hypertension [25], dyslipidemia [26], medication use such as statin [27] or aspirin [28] as reported by a self-questionnaire, height, weight, body mass index (BMI, kg/m^2^) [29], waist circumference, and blood tests including markers of diabetes, dyslipidemia, inflammation [30], dehydration [31], iron deficiency [32], and viral infection [33, 34].

Daily exercise duration was categorized into 3 subgroups. According to 2013 AHA/ACC guideline [35], 40-min exercise per day was suggested to affect blood pressure and cholesterol. Therefore, we divided low and high amount of daily exercise duration by 40 min. Blood tests including white blood cell count, red blood cell count, platelet count, sodium, potassium, fasting glucose, hemoglobin A1c, total cholesterol, triglycerides, high-density lipoprotein (HDL) cholesterol, low-density lipoprotein (LDL) cholesterol, hemoglobin, and total iron binding capacity (TIBC) were performed. Blood samples were collected after at least 10 h of fasting. The Department of Laboratory Medicine and Genetics at Samsung Medical Center has participated in several proficiency testing programs operated by the Korean Association of Quality Assurance for Clinical Laboratory, the Asian Network of Clinical Laboratory Standardization and Harmonization, and the College of American Pathologists.

### Pentacam nuclear staging

**Fig. 1:**
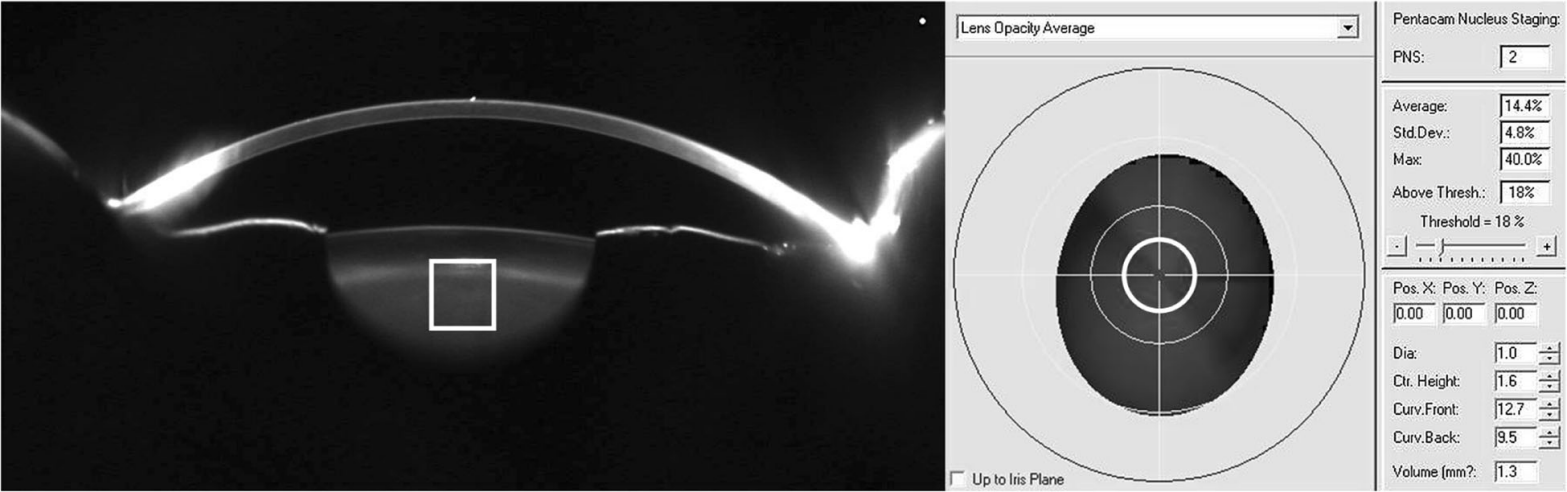
Pentacam Nucleus Staging (PNS) Measurement (PNS score = 2; average Scheimpflug lens density = 14.4%, maximum Scheimpflug lens density = 40.0%)

Quantitative lens density was measured with Pentacam device, as in the previous report [4]. All images were obtained using the same device and consistent environment after equipment calibration. Participants without pupil dilation placed their chins on a chin rest. The Scheimpflug image of each eye was manually focused and centered. Built-in densitometry software automatically measured PNS measurements (Fig. 1). To increase the reliability of the data, Scheimpflug images of the medical examination included in the study were verified by the ophthalmologist, and ophthalmologist confirmed that PNS measurements were obtained at the nucleus position of lens. The grading scores from 0 to 5, and the average and maximum value of nuclear density from 0 to 100% were recorded.

Presence of nuclear cataract was defined by corrected visual acuity and PNS measurements. According to our previous criteria [4], presenile nuclear cataract was diagnosed when the log MAR visual acuity with correction was greater than or equal to 0.2 [4] and one or more of the following were met: PNS grading score ≥ 1, average value of nuclear density ≥ 15%, maximum value of nuclear density ≥ 30%.

### Statistical analysis

Between-group differences (normal group vs. presenile nuclear cataract group) were evaluated using Wilcoxon test for continuous variables, chi-squared test for categorical variables, and Fisher’s exact test for analysis of factors with small counts of ≤5. To identify risk factors for presenile nuclear cataract, generalized estimation equation (GEE) models were constructed by considering paired eyed data. In the first step, univariate GEE was performed to identify possible prognostic factors. In the second step, multivariate GEE was performed using variables with *p* values less than 0.2 on univariate GEE to refine the predictive model. All statistical tests were two-tailed and performed with *p* < 0.2 considered statistically significant in univariate analysis, and *p* < 0.05 considered statistically significant in multivariate analysis. Distributions for continuous variables are expressed as means ± standard deviations (SDs). Statistical analysis was performed using SAS version 9.4 (SAS Institute, Cary, NC).

## Results

### Descriptive characteristics

A total of 532 eyes of 266 participants were included in this study. Seventy-four eyes (13.91%) were diagnosed with presenile nuclear cataract by PNS. The age of subjects was 45.12 ± 3.78 years (range, 31–49), and 183 participants (68.80%) of the total analyzed population were men. The log MAR visual acuity with correction was 0.07 ± 0.17, and PNS measurements were as follows; PNS score = 0.52 ± 0.54, average Scheimpflug lens density = 10.72 ± 1.55%, maximum Scheimpflug lens density = 38.05 ± 16.22%.

Descriptive characteristics and between-group differences were analyzed in Table 1. 32.24% of participants were current smokers; 2.44% of participants reported daily alcohol intake; 9.72% of participants performed no physical exercise, 14.66% of participants had hypertension, 6.02% of participants had diabetes, 16.92% of participants had dyslipidemia, 1.88% of participants had asthma, 4.14% of participants had tuberculosis, and 4.14% of participants showed hepatitis B virus (HBV) seropositivity.

**Table 1:** Demographic characteristics and between-group analysis of participants

	Total (*n* = 266)	Normal (*n* = 192)	Presenile nuclear cataract (*n* = 74)^d^	*p* value
Age (years)	45.12 ± 3.78 (*N* = 266)	45.26 ± 3.65 (*N* = 192)	44.76 ± 4.12 (*N* = 74)	0.471^a^
Sex, male	183/266 (68.8%)	136/192 (72.18%)	47/74 (63.51%)	0.248^b^
Smoking status				0.306^b^
Never	116/245 (47.35%)	86/174 (49.43%)	30/71 (42.25%)	
Former (quit ≥1 year ago)	50/245 (20.41%)	37/174 (21.26%)	13/71 (18.31%)	
Current	79/245 (32.24%)	51/174 (29.31%)	28/71 (39.44%)	
Age at starting smoking (years)	20.11 ± 3.99 (*N* = 131)	20.45 ± 4.09 (*N* = 92)	19.33 ± 3.69 (*N* = 39)	0.210^a^
Smoking duration (years)	21.30 ± 7.81 (*N* = 121)	20.93 ± 7.88 (*N* = 85)	22.17 ± 7.70 (*N* = 36)	0.448^a^
Smoking cigarettes per day				0.366^a^
≤ 10 cigarettes	38/126 (30.16%)	29/88 (32.95%)	9/38 (23.68%)	
11–20 cigarettes	48/126 (38.10%)	34/88 (38.64%)	14/38 (36.84%)	
21–30 cigarettes	33/126 (26.19%)	22/88 (25.00%)	11/38 (28.95%)	
≥ 30 cigarettes	7/126 (5.56%)	3/88 (3.41%)	4/38 (10.53%)	
Alcohol consumption status				0.456^b^
Never	53/261 (20.31%)	36/188 (19.15%)	17/73 (23.29%)	
Ever	208/261 (79.69%)	152/188 (80.85%)	56/73 (76.71%)	
Alcohol consumption duration (years)	23.07 ± 7.07 (*N* = 192)	23.04 ± 7.20 (*N* = 141)	23.14 ± 6.78 (*N* = 51)	0.734^a^
Alcohol consumption frequency				0.532^c^
≤ 1 day/month	24/205 (11.71%)	16/149 (10.74%)	8/56 (14.29%)	
2–3 days/month	63/205 (30.73%)	46/149 (30.87%)	17/56 (30.36%)	
1–2 days/week	63/205 (30.73%)	49/149 (32.89%)	14/56 (25.00%)	
3–4 days/week	41/205 (20.00%)	29/149 (19.46%)	12/56 (21.43%)	
5–6 days/week	9/205 (4.39%)	7/149 (4.70%)	2/56 (3.57%)	
Everyday	5/205 (2.44%)	2/149 (1.34%)	3/56 (5.36%)	
Alcohol consumption amount (Units/time)				0.940^b^
1~ 2	56/203 (27.59%)	39/147 (26.53%)	17/56 (30.36%)	
3~ 6	48/203 (23.65%)	36/147 (24.49%)	12/56 (21.43%)	
7~ 13	76/203 (37.44%)	55/147 (37.41%)	21/56 (37.50%)	
≥ 14	23/203 (11.33%)	17/147 (11.56%)	6/56 (10.71%)	
Physical exercise degree, *n* (%)				0.558^b^
Almost absent	26/253 (10.28%)	20/182 (10.99%)	6/71 (8.45%)	
Mild	67/253 (26.48%)	47/182 (25.82%)	20/71 (28.17%)	
Moderate	134/253 (52.96%)	99/182 (54.40%)	35/71 (49.30%)	
Vigorous	26/253 (10.28%)	16/182 (8.79%)	10/71 (14.08%)	
Physical exercise frequency, *n* (%)				0.324^b^
None	27/245 (11.02%)	18/178 (10.11%)	9/67 (13.43%)	
1–2 days/week	99/245 (40.41%)	75/178 (42.13%)	24/67 (35.82%)	
3–4 days/week	79/245 (32.24%)	60/178 (33.71%)	19/67 (28.36%)	
≥ 5 days/week	40/245 (16.33%)	25/178 (14.04%)	15/67 (22.39%)	
Daily exercise duration, *n* (%)				0.065^b^
Low amounts (1–40 min/day)	105/247 (42.51%)	83/178 (46.63%)	22/69 (31.88%)	
None	24/247 (9.72%)	14/178 (7.87%)	10/69 (14.49%)	
High amounts (≥41 min/day)	118/247 (47.77%)	81/178 (45.51%)	37/69 (53.62%)	
Height (cm)	169.66 ± 8.55 (*N* = 266)	169.35 ± 8.39 (*N* = 192)	170.45 ± 8.98 (*N* = 74)	0.349^a^
Weight (kg)	72.50 ± 16.27 (*N* = 266)	72.34 ± 16.43 (*N* = 192)	72.92 ± 15.96 (*N* = 74)	0.793^a^
BMI (kg/m^2^)	24.98 ± 4.23 (*N* = 266)	25.01 ± 4.29 (*N* = 192)	24.90 ± 4.12 (*N* = 74)	0.923^a^
WHR	0.91 ± 0.07 (*N* = 266)	0.91 ± 0.07 (*N* = 192)	0.92 ± 0.08 (*N* = 74)	0.586^a^
Hypertension, *n* (%)	39/266 (14.66%)	30/192 (15.63%)	9/74 (12.16%)	0.474^b^
Diabetes, *n* (%)	16/266 (6.02%)	14/192 (7.29%)	2/74 (2.70%)	0.249^c^
Dyslipidemia, *n* (%)	45/266 (16.92%)	31/192 (16.15%)	14/74 (18.92%)	0.589^b^
Asthma, *n* (%)	5/266 (1.88%)	2/192 (1.04%)	3/74 (4.05%)	0.133^b^
Tuberculosis, *n* (%)	11/266 (4.14%)	5/192 (2.60%)	6/74 (8.11%)	0.078^b^
Thyroid disease, *n* (%)	8/266 (3.01%)	7/192 (3.65%)	1/74 (1.35%)	0.450^c^
HbA1c (%)	5.58 ± 0.68 (*N* = 256)	5.62 ± 0.77 (*N* = 183)	5.48 ± 0.36 (*N* = 73)	0.511^a^
Glucose (mg/dL)	95.89 ± 21.91 (*N* = 266)	97.48 ± 24.71 (*N* = 192)	91.74 ± 11.00 (*N* = 74)	0.058^a^
Cholesterol (mg/dL)	197.09 ± 35.88 (*N* = 266)	198.67 ± 35.65 (*N* = 192)	192.99 ± 36.41 (*N* = 74)	0.257^a^
HDL cholesterol (mg/dL)	58.65 ± 17.79 (*N* = 265)	57.92 ± 15.41 (*N* = 191)	60.53 ± 22.84 (*N* = 74)	0.578^a^
LDL cholesterol (mg/dL)	125.69 ± 32.18 (*N* = 265)	127.09 ± 31.64 (*N* = 191)	122.07 ± 33.49 (*N* = 74)	0.312^a^
Triglyceride (mg/dL)	126.50 ± 127.86 (*N* = 265)	133.31 ± 143.60 (*N* = 191)	108.92 ± 70.87 (*N* = 74)	0.097^a^
WBC (/mm^3^)	5763.8 ± 1761.3 (*N* = 266)	5906.8 ± 1872.3 (*N* = 192)	5392.7 ± 1377.2 (*N* = 74)	0.074^a^
CRP (mg/dL)	0.20 ± 0.51 (*N* = 247)	0.18 ± 0.41 (*N* = 177)	0.24 ± 0.72 (*N* = 70)	0.235^a^
Sodium (mmol/L)	141.66 ± 1.83 (*N* = 245)	141.63 ± 1.87 (*N* = 176)	141.74 ± 1.75 (*N* = 69)	0.734^a^
Potassium (mmol/L)	4.24 ± 0.31 (*N* = 245)	4.24 ± 0.33 (*N* = 176)	4.23 ± 0.26 (*N* = 69)	0.966^a^
Hemoglobin (g/dL)	14.73 ± 1.79 (*N* = 266)	14.78 ± 1.75 (*N* = 192)	14.60 ± 1.91 (*N* = 74)	0.476^a^
TIBC (μg/dL)	324.66 ± 44.73 (*N* = 245)	321.31 ± 44.96 (*N* = 176)	333.20 ± 43.28 (*N* = 69)	0.040^a^
HBV seropositive, *n* (%)	11/266 (4.14%)	6/192 (3.13%)	5/74 (6.76%)	0.186^a^
HCV seropositive, *n* (%)	3/266 (1.13%)	2/192 (1.04%)	1/74 (1.35%)	1.000^a^
Medication history of aspirin	17/266 (6.39%)	13/192 (6.77%)	4/74 (5.41%)	0.787^b^
Medication history of statin	20/266 (7.52%)	17/192 (8.85%)	3/74 (4.05%)	0.183^b^
Medication history of nutritional supplements	39/266 (14.66%)	30/192 (15.63%)	9/74 (12.16%)	0.474^b^

^a^Wilcoxon rank sum test, ^b^Chi-squared test, ^c^Fisher’s exact test

^d^If at least one eye was diagnosed as presenile nuclear cataract, it was considered a presenile nuclear cataract patient

Examples of physical exercise degree: almost absent (walking for less than 10 min), mild (walking, golf, housework), moderate (bicycle, fast walking, tennis, swimming, hiking), vigorous (aerobics, jogging, soccer)

Alcohol consumption units: 10 g of alcohol

*BMI* body mass index, *WHR* waist-hip ratio, *HbA1c* hemoglobin A1c, *HDL* high-density lipoprotein, *LDL* low-density lipoprotein, *CRP* C-reactive protein, *TIBC* total iron binding capacity, *HBV* hepatitis B virus, *HCV* hepatitis C virus

Values are either absolute values or mean ± standard deviation values

### Univariate analysis

The univariate GEE analysis for each factor is reported in Additional file 1: Table S1. Variables with a *p* value less than 0.2 on univariate GEE were considered possible risk factors for presenile nuclear cataract. Compared to normal group, presenile nuclear cataract group showed higher proportion of current smokers (odds ratio (OR) = 1.72, 95% confidence interval (CI), 0.97–3.08, *p* = 0.0656), younger age at starting smoking (OR = 0.90, 95% CI, 0.80–1.01, *p* = 0.0791), longer smoking duration (OR = 1.04, 95% CI, 0.99–1.09, *p* = 0.1636), smoked over 30 cigarettes per day (OR = 2.13, 95% CI, 0.67–6.79, *p* = 0.1998), no physical exercise and a high amount of physical exercise (OR = 2.45, 95% CI, 1.08–5.58, *p* = 0.0322; OR = 1.97, 95% CI, 1.10–3.52, *p* = 0.0223), taller (OR = 1.02, 95% CI, 0.99–1.05, *p* = 0.1520), asthma (OR = 4.87, 95% CI, 0.99–23.84, *p* = 0.0510), tuberculosis (OR = 3.30, 95% CI, 1.09–9.97, *p* = 0.0341), lower hemoglobin A1c (OR = 0.71, 95% CI, 0.45–1.13, *p* = 0.1509), lower glucose level (OR = 0.99, 95% CI, 0.97–1.00, *p* = 0.1015), higher TIBC (OR = 1.00, 95% CI, 1.00–1.01, *p* = 0.0533), and higher HBV seropositivity (OR = 2.82, 95% CI, 0.91–8.74, *p* = 0.0729).

The association with presenile nuclear cataract was not significantly different with age (OR = 0.97, 95% CI, 0.91–1.04, *p* = 0.3422) and sex (OR = 1.24, 95% CI, 0.73–2.10, *p* = 0.4223). Other descriptive characteristics were comparable between normal and presenile nuclear cataract groups. There was no significant association between presenile nuclear cataract and other modifiable lifestyle-related risk factors, such as alcohol consumption, weight, BMI, and waist-hip ratio (WHR, *p* > 0.2). Cholesterol profile including total cholesterol, HDL, LDL, and triglyceride did not show a significant association with nuclear cataract (*p* > 0.2).

### Multivariate analysis

The results of multivariate GEE analysis adjusted for variables with a *p* value less than 0.2 on univariate analysis are reported in Table 2. Data regarding smoking, alcohol consumption, and physical exercise were collected using various survey questions. Variables in these questionnaire items were then adjusted by considering clinical and statistical significance. Smoking status, alcohol consumption frequency, and daily physical exercise duration were analyzed.

**Table 2:** Risk factors for presenile nuclear cataract, as estimated from univariable and multivariable generalized estimation equation

Risk factors	Univariate analysis		Multivariate analysis	
	OR (95% CI)	*p* value	OR (95% CI)	*p* value
Smoking status		0.1894		0.1296
Never	Reference		Reference	
Former (quit ≥1 year ago)	1.04 (0.51–2.12)	0.9206	1.83 (0.74–4.51)	0.1882
Current	1.72 (0.97–3.08)	0.0656	2.80 (1.10–7.12)	0.0310
Alcohol consumption frequency		0.7793		0.9150
≤ 1 day/month	Reference		Reference	
2–3 days/month	1.04 (0.44–2.46)	0.9381	1.43 (0.42–4.85)	0.5662
1–2 days/week	0.91 (0.37–2.23)	0.8367	0.83 (0.26–2.61)	0.7505
3–4 days/week	1.34 (0.52–3.43)	0.5409	1.00 (0.27–3.73)	0.9951
5–6 days/week	0.64 (0.14–2.97)	0.5691	0.81 (0.13–4.95)	0.8205
Everyday	2.20 (0.64–7.49)	0.2087	1.00 (0.24–4.20)	0.9970
Daily exercise duration, *n* (%)		0.0295		0.0107
Low amounts (1-40 min/day)	Reference		Reference	
None	2.45 (1.08–5.58)	0.0322	3.99 (1.27–12.52)	0.0178
High amounts (≥41 min/day)	1.97 (1.10–3.52)	0.0223	2.92 (1.38–6.22)	0.0053
Height (cm)	1.02 (0.99–1.05)	0.1520	1.04 (1.00–1.10)	0.0808
Asthma	4.87 (0.99–23.84)	0.0510	8.93 (1.12–71.15)	0.0386
Tuberculosis	3.30 (1.09–9.97)	0.0341	4.28 (1.36–13.50)	0.0131
HbA1c (%)	0.71 (0.45–1.13)	0.1509	0.70 (0.38–1.28)	0.2508
TIBC (μg/dL)	1.01 (1.00–1.01)	0.0533	1.01 (1.00–1.02)	0.0059
HBV seropositive	2.82 (0.91–8.74)	0.0729	2.98 (0.82–10.89)	0.0979

*OR* odds ratio, *CI* confidence interval, *HbA1c* hemoglobin A1c, *TIBC* total iron binding capacity, *HBV* hepatitis B virus

The multivariate model identified current smoker (OR = 2.80, 95% CI, 1.10–7.12, *p* = 0.0310), non-exercise and high amount of physical exercise (OR = 3.99, 95% CI, 1.27–12.52, *p* = 0.0178; OR = 2.92, 95% CI, 1.38–6.22, *p* = 0.0053), asthma (OR = 8.93, 95% CI, 1.12–71.15, *p* = 0.0386), tuberculosis (OR = 4.28, 95% CI, 1.36–13.50, *p* = 0.0131), and higher TIBC (OR = 1.01, 95% CI, 1.00–1.02, *p* = 0.0059) as predictive of presenile nuclear cataract. Alcohol consumption frequency, height, hemoglobin A1c, and HBV seropositivity did not show significant associations with nuclear cataract (*p* > 0.05).

## Discussion

Detection of early nuclear cataract is important in clinical and preventive medicine [36]. Relatively high incidence of presenile nuclear cataract (13.91%) in the current study indicates that health screening exams are important in early diagnosis of presenile nuclear cataract. Pentacam detects early nuclear cataract and quantitatively measures the severity of nuclear cataract [6]. Health screening data allows a large sample size and less biased selection of participants without significant ophthalmologic problems as the normal population. Therefore, risk factors for presenile nuclear cataract were identified using Pentacam and a health screening test in this study.

In the present study, current smokers showed a higher risk than never smokers (OR = 2.80, 95% CI, 1.10–7.12, *p* = 0.0310), consistent with previous reports [18, 37]. Ye et al. reported that smoking is a significant risk factor for nuclear cataract [18]. Copper, cadmium, and lead concentrations in crystalline lenses were higher in cataract patients, and this could be related with smoking [38]. In the present study, early smoking age, smoking duration, and heavy smoking (≥ 30 cigarettes/day) showed significant effects on univariate analysis (*p* < 0.2) and could also affect formation of nuclear cataract. Therefore, smoking prevention and cessation are important in preventing formation of nuclear cataract.

In the current study, daily physical exercise duration and presenile nuclear cataract showed a U-shaped relationship. Participants with low amount of exercise (1–40 min/day) showed the lowest nuclear cataract formation. Participants that were non-exerciser (OR = 3.99, 95% CI, 1.27–12.52, *p* = 0.0178) or who had a high amount (≥41 min/day) of exercise (OR = 2.92, 95% CI, 1.38–6.22, *p* = 0.0053) showed more nuclear cataract formation. Non-exercise group showed larger proportion co-morbidity than any amount of exercise group such as stroke history (4.2% vs 2.2%) in this study. Therefore, we should consider the possibility that co-morbidity has affected cataract formation [39]. Zheng et al. reported that Leisure time inactivity (< 1 h/day) was associated with increased risk of cataract [20]. However, they did not perform sub-group analysis of non-exerciser and any amount of exercise group, especially less than an hour daily. High amounts of physical exercise group showed variable incidence of presenile nuclear cataract in previous study [40]. High amounts of physical exercise group could create more free radicals [41] and could be exposed high amount of ultraviolet radiation during outdoor activity. However, high amounts of physical exercise also reduce glucose levels and are correlated to a healthy life style. Therefore, to eliminate the effect of these confounding variables for physical exercise, further study is required.

Asthma was a significant risk factor for presenile nuclear cataract in this study (OR = 8.93, 95% CI, 1.12–71.15, *p* = 0.0386). Asthma patients may receive systemic steroid treatments, which likely explain the higher incidence of cataract [22]. However, because the current study used health screening data, only a small number of participants with asthma were included in the study. Also, information on steroid treatment history was not recorded. Additional study is necessary to reveal the relationship between asthma and nuclear cataract formation.

Tuberculosis is also a significant risk factor of presenile nuclear cataract in this study (OR = 4.28, 95% CI, 1.36–13.50, *p* = 0.0131). The possible mechanism of cataract formation with tuberculosis was suggested in a previous report [24]. The mechanism is likely complicated and includes direct and indirect biological effects of tuberculosis, steroid treatments, toxicity of anti-tuberculosis treatments, and low socioeconomic status [24]. Also, only a small number of participants with tuberculosis were included, and the confidence interval was wide because this study is based on health screening. Further study is needed to reveal the relationship of tuberculosis and nuclear cataract formation.

Higher TIBC level is related to iron deficiency [42]. Iron deficiency is related to cataract [43], and iron supplementation may reduce oxidative stress [44]. In the current study, higher TIBC was a significant risk factor for cataract (OR = 1.01, 95% CI, 1.00–1.02, *p* = 0.0059).

Age is a strong risk factor for nuclear cataract [14]. However, in this study, age was not significantly related to nuclear cataract. Most previous studies regarding nuclear cataract did not include presenile age under 50 or did not analyze the association with age due to low incidence of presenile nuclear cataract. Klein et al. included participants 43 to 83 years old and showed nuclear cataract prevalence of 12.4% in the 43 to 54 years old age group. However, they did not perform subgroup analysis of this group in the Beaver Dam Eye Study [45]. Foster et al. included participants 40 to 81 years old and showed a significant association between age and nuclear cataract (OR = 5.6, 95% CI. 4.6–6.8, *p* < 0.001), but they did not perform subgroup analysis of this group in the Tanjong Pagar Survey [46]. Therefore, there is currently insufficient evidence for age as a risk factor of nuclear cataract in the presenile age group. Another explanation for the lack of association with age is sampling bias of the current study. Patients with severe cataract in older age received early cataract surgery and were excluded from this study.

Alcohol showed conflicting results with cataract in a previous study [47]. In the current study, alcohol did not show any association with nuclear cataract formation in status, duration, amount, or frequency. Alcohol could be a confounder of age, diet, smoking, and socioeconomic status. Prospective study is necessary to reveal the effect of alcohol on presenile nuclear cataract. In the current study, blood glucose and hemoglobin A1c level did not show relationships with nuclear cataract formation. Although diabetes is an important risk factor for cataract, diabetes shows conflicting results in nuclear type [48]. Furthermore, data from the health screening test causes selection bias from the healthy user effect. Participants who received health screening may have a healthy life style and low possibility of diabetes.

There were several studies about risk factors of age-related cataract. According to Taizhou Eye Study, age-related cataract is related with older age, increased outdoor activity, lower education level, female, no outdoor eye protection, low high-density lipoprotein, high low-density lipoprotein, high myopia, and increased pickled food intake [49]. In Los Angeles Latino Eye Study, older age, smoking, and myopia were related with age-related cataract. These studies included senile age group and used slit lamp examination for qualitative cataract grading [50]. In our study, smoking and amount of physical exercise were related with presenile nuclear cataract. We revealed relationship of smoking and nuclear cataract with detailed smoking questionnaire, and U-shaped relationship of daily physical exercise duration and nuclear cataract. High incidence of presenile nuclear cataract in high amounts of exercise group might not only be related with free radical, but also ultraviolet radiation while outdoor activity. Unfortunately, the questionnaires used in this study did not distinguish between indoor and outdoor activity. In addition, high incidence of presenile nuclear cataract in non-exercise group might be related with co-morbidity such as stroke [39].

Our study has several strengths, including its large sample size and detailed information on a number of potential risk factors for presenile nuclear cataract. Previously, few studies have investigated risk factors for presenile nuclear cataract because of relatively low incidence of this disease and absence of control data in the clinical setting. However, Pentacam method provides objective measurements to evaluate cataracts, especially in health examination. Our study has a large sample size and accurate detailed information for both cases and controls, providing helpful results that current smoking, asthma, tuberculosis, and iron deficiency status are risk factors for presenile nuclear cataract. In addition, the non-exerciser and high amount of daily physical exercise duration is risk factors for presenile nuclear cataract.

However, the current study is limited by its cross sectional nature using health screening data. In a cross-sectional study, the temporal relationship cannot be clearly defined, and it is difficult to establish causal inferences. There may also have been selection bias and healthy user bias. Also, confounding variables could not be perfectly excluded. Information on socioeconomic status, occupation, and residence area, which could be related to cataract formation, was not obtained. In addition, without pupil dilation, the accuracy of PNS score might be reduced. However, non-dilated PNS score could be a good screening tool for presenile nuclear cataract in general health examination setting which pupil dilatation is difficult.

## Conclusion

Presenile nuclear cataract is also related to current smoking, non-exercise and high amounts of physical exercise, asthma, tuberculosis, and iron deficiency status. Patients with asthma, tuberculosis, and iron deficiency anemia who complain of visual discomfort should receive frequent ophthalmic examinations to detect cataract and other eye problems.

## Additional file

Additional file 1: **Table S1.** Univariate Analyses using Generalized Estimation Equation for Presenile Nuclear Cataract using Various Risk Factors. Univariate analyses of presenile nuclear cataract and risk factors. (DOCX 38 kb)
