# Correction to: Risk factors of presenile nuclear cataract in health screening study

**DOI:** 10.1186/s12886-018-0972-2

**Published:** 2018-11-30

**Authors:** Seung Wan Nam, Dong Hui Lim, Kyu Yeon Cho, Hye Seung Kim, Kyunga Kim, Tae-Young Chung

**Affiliations:** 1 Department of Ophthalmology, Samsung Medical Center, Sungkyunkwan University School of Medicine, Seoul, South Korea; 2 Department of Preventive Medicine, Graduate School, The Catholic University of Korea, Seoul, South Korea; 3 Biostatistics and Clinical Epidemiology Center, Research Institute for Future Medicine, Samsung Medical Center, Seoul, South Korea

## Correction

Following publication of the original article [1], the authors notified us of that due to miscommunication during proofing their given names were not presented in their full form, but only using the initials.

The original publication has been corrected. The initial and corrected names are presented below.

- Author names as originally published:

SW Nam, DH Lim, KY Cho, HS Kim, K Kim and T-Y Chung

- Author names corrected:

Seung Wan Nam, Dong Hui Lim, Kyu Yeon Cho, Hye Seung Kim, Kyunga Kim and Tae-Young Chung
