# Supplementary material for: Risk factors of presenile nuclear cataract in health screening study
Source: BMC Ophthalmol. 2018 Oct 11;18:263. doi: 10.1186/s12886-018-0928-6 (PMC6180395; doi:10.1186/s12886-018-0928-6)
Supplement: Supplementary file 1 — Table S1. Univariate Analyses using Generalized Estimation Equation for Presenile Nuclear Cataract using Various Risk Factors. Univariate analyses of presenile nuclear cataract and risk factors. (DOCX 38 kb) [file 12886_2018_928_MOESM1_ESM.docx]

**Table S1. Univariate Analyses using Generalized Estimation Equation for Presenile Nuclear Cataract using Various Risk Factors**

| Risk factors | Cases, n (%) | OR (95% CI) | *p* value |  |
| --- | --- | --- | --- | --- |
| Age |  | 0.97 (0.91-1.04) | 0.3422 |  |
| Sex, male |  | 1.24 (0.73-2.10) | 0.4223 |  |
| Smoking status | 490 |  |  |  |
| Never | 232 (47.35%) | Reference |  |  |
| Former (quit ≥ 1 year ago) | 100 (20.41%) | 1.04 (0.51-2.12) | 0.9206 |  |
| Current | 158 (32.24%) | 1.72 (0.97-3.08) | 0.0656 |  |
| Age at starting smoking (years) |  | 0.90 (0.80-1.01) | 0.0791 |  |
| Smoking duration (years) |  | 1.04 (0.99-1.09) | 0.1636 |  |
| Smoking cigarettes per day | 252 |  |  |  |
| ≤10 cigarettes | 76 (30.16%) | Reference |  |  |
| 11-20 cigarettes | 96 (38.10%) | 1.40 (0.54-3.62) | 0.4831 |  |
| 21-30 cigarettes | 66 (26.19%) | 1.44 (0.53-3.86) | 0.4735 |  |
| ≥30 cigarettes | 14 (5.56%) | 2.13 (0.67-6.79) | 0.1998 |  |
| Alcohol consumption status | 522 |  |  |  |
| Never | 106 (20.31%) | Reference |  |  |
| Ever | 416 (79.69%) | 0.73 (0.04-1.35) | 0.3176 |  |
| Alcohol consumption duration (years) |  | 1.01 (0.96-1.05) | 0.7873 |  |
| Alcohol consumption frequency | 410 |  |  |  |
| ≤1 day/month | 49 (11.95%) | Reference |  |  |
| 2-3 days/month | 125 (30.49%) | 1.04 (0.44-2.46) | 0.9381 |  |
| 1-2 days/week | 126 (30.73%) | 0.91 (0.37-2.23) | 0.8367 |  |
| 3-4 days/week | 82 (20.00%) | 1.34 (0.52-3.43) | 0.5409 |  |
| 5-6 days/week | 18 (4.39%) | 0.64 (0.14-2.97) | 0.5691 |  |
| Everyday | 10 (2.44%) | 2.20 (0.64-7.49) | 0.2087 |  |
| Alcohol consumption amount (Units/time) | 406 |  |  |  |
| 1~2 | 112 (27.59%) | Reference |  |  |
| 3~6 | 96 (23.65%) | 0.72 (0.33-1.58) | 0.4135 |  |
| 7~13 | 152 (37.44%) | 1.04 (0.51-2.11) | 0.9163 |  |
| ≥14 | 46 (11.33%) | 1.12 (0.39-3.21) | 0.8344 |  |
| Physical exercise degree, n (%) | 506 |  |  |  |
| Almost absent | 52 (10.28%) | Reference |  |  |
| Mild | 133 (26.28%) | 1.40 (0.50-3.91) | 0.5194 |  |
| Moderate | 269 (53.16%) | 1.05 (0.40-2.75) | 0.9266 |  |
| Vigorous | 52 (10.28%) | 1.83 (0.59-5.75) | 0.2983 |  |
| Physical exercise frequency, n (%) | 490 |  |  |  |
| None | 54 (11.02%) | Reference |  |  |
| 1-2 days/week | 198 (40.41%) | 0.67 (0.29-1.57) | 0.3557 |  |
| 3-4 days/week | 158 (32.24%) | 0.74 (0.30-1.78) | 0.4944 |  |
| ≥5 days/week | 80 (16.33%) | 1.22 (0.48-3.08) | 0.6777 |  |
| Daily exercise duration, n (%) | 494 |  |  |  |
| Low amounts (1-40 min/day) | 209 (42.31%) | Reference |  |  |
| None | 48 (9.72%) | 2.45 (1.08-5.58) | 0.0322 |  |
| High amounts (≥41 min/day) | 237 (47.98%) | 1.97 (1.10-3.52) | 0.0223 |  |
| Height (cm) |  | 1.02 (0.99-1.05) | 0.1520 |  |
| Weight (kg) |  | 1.00 (0.99-1.02) | 0.6071 |  |
| BMI (kg/m^2^) |  | 1.00 (0.93-1.06) | 0.8690 |  |
| WHR |  | 8.31 (0.17-418.40) | 0.2898 |  |
| Hypertension, n (%) |  | 0.81 (0.37-1.77) | 0.5967 |  |
| Diabetes, n (%) |  | 0.47 (0.01-2.17) | 0.3298 |  |
| Dyslipidemia, n (%) |  | 1.01 (0.54-1.90) | 0.9778 |  |
| Asthma, n (%) |  | 4.87 (0.99-23.84) | 0.0510 |  |
| Tuberculosis, n (%) |  | 3.30 (1.09-9.97) | 0.0341 |  |
| HbA1c (%) |  | 0.71 (0.45-1.13) | 0.1509 |  |
| Glucose (mg/dL) |  | 0.99 (0.97-1.00) | 0.1015 |  |
| Cholesterol (mg/dL) |  | 1.00 (0.99-1.00) | 0.2380 |  |
| HDL cholesterol (mg/dL) |  | 1.01 (0.99-1.02) | 0.3245 |  |
| LDL cholesterol (mg/dL) |  | 1.00 (0.99-1.00) | 0.2029 |  |
| Triglyceride (mg/dL) |  | 1.00 (0.99-1.00) | 0.2095 |  |
| CRP (mg/dL) |  | 1.07 (0.75-1.53) | 0.7192 |  |
| Sodium (mmol/L) |  | 1.05 (0.91-1.22) | 0.4878 |  |
| Potassium (mmol/L) |  | 1.07 (0.51-2.25) | 0.8523 |  |
| Hemoglobin (g/dL) |  | 0.97 (0.85-1.12) | 0.7208 |  |
| TIBC (μg/dL) |  | 1.00 (1.00-1.01) | 0.0533 |  |
| HBV seropositive, n (%) |  | 2.82 (0.91-8.74) | 0.0729 |  |
| HCV seropositive, n (%) |  | 0.93 (0.13-6.46) | 0.9425 |  |
| Medication history of aspirin |  | 1.23 (0.39-3.87) | 0.7299 |  |
| Medication history of statin |  | 0.65 (0.18-2.31) | 0.5027 |  |
| Medication history of nutritional supplements |  | 0.83 (0.38-1.80) | 0.6280 |  |

Examples of physical exercise degree: almost absent (walking for less than 10 min), mild (walking, golf, housework), moderate (bicycle, fast walking, tennis, swimming, hiking), vigorous (aerobics, jogging, soccer).

Alcohol consumption units: 10 g of alcohol

OR: odds ratio, CI: confidence interval, BMI: body mass index, WHR: waist-hip ratio, HbA1c: hemoglobin A1c, HDL: high-density lipoprotein, LDL: low-density lipoprotein, CRP: C-reactive protein, TIBC: total iron binding capacity, HBV: hepatitis B virus, HCV: hepatitis C virus
